# Combined genomic, transcriptomic, and metabolomic analyses provide insights into the fruit development of bottle gourd (*Lagenaria siceraria*)

**DOI:** 10.1093/hr/uhae335

**Published:** 2024-11-27

**Authors:** Xuelian He, Yanyan Zheng, Songguang Yang, Ying Wang, Yu'e Lin, Biao Jiang, Dasen Xie, Wenrui Liu, Qingwu Peng, Jinhua Zuo, Min Wang

**Affiliations:** Institute of Agri-food Processing and Nutrition, Beijing Academy of Agricultural and Forestry Sciences, Beijing Key Laboratory of Fruits and Vegetable Storage and Processing, Key Laboratory of Vegetable Postharvest Processing of Ministry of Agriculture and Rural Areas, State Key Laboratory of Vegetable Biobreeding, Beijing Vegetable Research Center, Beijing Academy of Agriculture and Forestry Science, Beijing 100097, China; Beijing Engineering and Technology Research Center of Food Additives, Beijing Advanced Innovation Center for Food Nutrition and Human Health, School of Food and Health, Beijing Technology and Business University (BTBU), Beijing 100048, China; Institute of Agri-food Processing and Nutrition, Beijing Academy of Agricultural and Forestry Sciences, Beijing Key Laboratory of Fruits and Vegetable Storage and Processing, Key Laboratory of Vegetable Postharvest Processing of Ministry of Agriculture and Rural Areas, State Key Laboratory of Vegetable Biobreeding, Beijing Vegetable Research Center, Beijing Academy of Agriculture and Forestry Science, Beijing 100097, China; Vegetable Research Institute, Guangdong Academy of Agricultural Sciences/ Guangdong Key Laboratory for New Technology Research of Vegetables, Guangzhou 510640, China; Institute of Vegetables, Zhejiang Academy of Agricultural Sciences, Hangzhou 310012, China; Vegetable Research Institute, Guangdong Academy of Agricultural Sciences/ Guangdong Key Laboratory for New Technology Research of Vegetables, Guangzhou 510640, China; Vegetable Research Institute, Guangdong Academy of Agricultural Sciences/ Guangdong Key Laboratory for New Technology Research of Vegetables, Guangzhou 510640, China; Vegetable Research Institute, Guangdong Academy of Agricultural Sciences/ Guangdong Key Laboratory for New Technology Research of Vegetables, Guangzhou 510640, China; Vegetable Research Institute, Guangdong Academy of Agricultural Sciences/ Guangdong Key Laboratory for New Technology Research of Vegetables, Guangzhou 510640, China; Vegetable Research Institute, Guangdong Academy of Agricultural Sciences/ Guangdong Key Laboratory for New Technology Research of Vegetables, Guangzhou 510640, China; Institute of Agri-food Processing and Nutrition, Beijing Academy of Agricultural and Forestry Sciences, Beijing Key Laboratory of Fruits and Vegetable Storage and Processing, Key Laboratory of Vegetable Postharvest Processing of Ministry of Agriculture and Rural Areas, State Key Laboratory of Vegetable Biobreeding, Beijing Vegetable Research Center, Beijing Academy of Agriculture and Forestry Science, Beijing 100097, China; Vegetable Research Institute, Guangdong Academy of Agricultural Sciences/ Guangdong Key Laboratory for New Technology Research of Vegetables, Guangzhou 510640, China

## Abstract

Bottle gourd (*Lagenaria siceraria* (Molina) Standl) is a widely distributed Cucurbitaceae species, but gaps and low-quality assemblies have limited its genomic study. To address this, we assembled a nearly complete, high-quality genome of the bottle gourd (Pugua) using PacBio HiFi sequencing and Hi-C correction. The genome, being 298.67 Mb long with a ContigN50 of 28.55 Mb, was identified to possess 11 chromosomes, 11 centromeres, 18 telomeres, and 24 439 predicted protein-coding genes; notably, gap-free telomere-to-telomere assembly was accomplished for seven chromosomes. Based on the Pugua genome, the transcriptomic and metabolomic combined analyses revealed that amino acids and lipids accumulate during the expansion stage, while sugars and terpenoids increase during ripening. GA4 and genes of the Aux/IAA family mediate fruit expansion and maturation, while cell wall remodeling is regulated by factors such as *XTHs, EXPs*, polyphenols, and alkaloids, contributing to environmental adaptation. *GGAT2* was positively correlated with glutamate, a source of umami, and *SUS5* and *SPS4* expression aligned with sucrose accumulation. This study provides a valuable genetic resource for bottle gourd research, enhancing the understanding of Cucurbitaceae evolution and supporting further studies on bottle gourd development, quality, and genetic improvement.

## Introduction

*Lagenaria siceraria* (Molina) Standl (*L. siceraria*) is an annual diploid climbing plant of the genus Calabash in the Cucurbitaceae family with the origin of bottle gourd traced back to sub-Saharan Africa [1]. Two subspecies, African *Lagenaria siceraria ssp. siceraria* and Asian *L. siceraria ssp. Asiatica* were domesticated independently in Africa and Asia over 10 000 years of cultivation history with significant differences in fruit characteristics such as skin color, shape, and texture [2]. At present, bottle gourd is widely distributed in India, China, Brazil, and various European countries as it contains rich bioactive substances, including vitamins, choline, terpenoids, and flavonoids, and has significant pharmacological potential in treating anxiety, depression, cancer, hyperlipidemia, and cytotoxicity, which is also the basis for the young bottle gourd often used as a medical-food vegetable [3, 4].

In recent years, researchers have conducted extensive exploration of the genomes of the Cucurbitaceae family. The telomere-to-telomere (T2T) genomes of pumpkins [5], watermelons [6], melons [7–9], cucumbers [10], bitter gourds [11], and *Gynostemma pentaphyllum* [12] have been successively released, providing high-quality genomic resources for Cucurbitaceae breeding and improvement work. Research on the bottle gourd still focuses on genetic improvement and analysis in aspects such as horticultural traits, agronomic performance, nutritional and medicinal components, and biotic and abiotic stress resistance [13]. As a vegetable widely cultivated and consumed globally, the attention paid to more detailed biological processes such as fruit development of the bottle gourd is extremely limited. In 2023, Buthelezi first reported the changes in buds during the development of bottle gourd and the relationship between the inflorescence stem and the fruit [14]. In 2024, a study was also published on changes in micronutrients and macronutrients in bottle gourd during development [15]. These studies provide limited information on the development of bottle gourd. Therefore, the molecular mechanism of bottle gourd development and ripening is still unclear, which hinders the development of bottle gourd quality improvement.

Lagenaria may be the only known Cucurbitaceae species present in both the early prehistoric New and Old Worlds [16]. The genome sequence of bottle gourd contains valuable genetic information regarding fruit shape genetic diversity, soft and sweet flesh, and high medicinal value. Analyzing and improving the genome assembly of bottle gourd is crucial for advancing Cucurbitaceae genetics and the molecular genetics of bottle gourd. In 2013, Illumina HiSeq sequencing technology was used to obtain the first data, containing 305 112 scaffolds of a bottle gourd population genome (Bottle_gourd) [17]. Subsequently, Wu *et al.* [18] utilized this technique to obtain the genome sequence (Lsi_v1.0) of a high-quality stock-type bottle gourd inbred line ‘USVLIVR-Ls’, providing new insights into Cucurbitaceae genome evolution and improvement. With the continuous progress of genome sequencing and assembly technology, Xu *et al.* [19] published the genome of the chromosome-level food-type Hangzhou Gourd (ZAAS_Lsic_2.0) in 2021. Compared with the previous short-read version, ZAAS_Lsic_2.0 is a long-read genome assembled using third-generation sequencing technology PacBio Sequel [17–19], significantly improving the continuity and integrity of the bottle gourd genome and advancing Cucurbitaceae genomics and genetic breeding research. However, many repeats, deletion sequences, and gaps still affect the identification of functional genes and the overall integrity of the genome, hindering functional genomics and breeding research for bottle gourd.

To achieve a high-quality bottle gourd genome with enhanced readability and integrity, and to further advance bottle gourd genetics research, a T2T genome of Pugua was assembled using PacBio HiFi, ONT ultra-long (100 k) extraction, and high-throughput chromatin capture Hi-C techniques. Additionally, the fundamental biological processes underlying the development of the Pugua fruit were delved by elucidating the differences in metabolic accumulation and gene expression patterns across various developmental stages. Our findings offer valuable genome resources for molecular research and contribute to our understanding of the metabolic regulation and genetic control mechanisms that govern fruit ontogeny in bottle gourd.

## Results

### Genomic characterization assessment

Pugua is a diploid karyotype. Based on the base distribution of 21.72 Gb Illumina sequencing data with a sequencing depth of 55.31×, the genome size is estimated to be approximately 297.40 Mb. The *K-mer* distribution analysis estimated the repetitive sequence content at 25.49% and revealed low heterozygosity, around .01% (Fig. S1A). These characteristics are conducive to constructing a fine genomic map.

### High-quality genome assembly

We employed multiple sequencing techniques to achieve a high-quality genome assembly. This included obtaining 16.03 Gb (~46× depth, 100.66 kb reads N50) of ONT ultra-long reads data, 30.99 Gb (~82× depth, 100.66 kb reads N50) of high-quality CCS data, and 39.60 Gb of Hi-C data (Table S1). Using these data, a 298.67 Mb genome sequence was generated through hifiasm assembly, Lachesis mounting, Hi-C error correction, auxiliary chromosome mounting, and redundancy assembly. Compared to the previously assembled ZAAS_Lsic_2.0, our genome assembly reduced the number of scaffolds and contigs from 27 and 71 to 11, respectively, with the contig N50 increasing from 11.21 to 28.55 Mb and contig N90 from 3.55 to 21.30 Mb (Table 1). Additionally, 53 gaps present in ZAAS_Lsic_2.0 were filled, resulting in a sequence length that accounts for 100% of the total localized chromosome sequence length**.**

**Table 1:** Comparison of bottle gourd genome assembly with previously published genomes.

Genome features	Pugua	ZAAS_Lsic_2.0^[19]^	Lsi_v1.0^[18]^	Bottle_gourd^a^
Cultivar	Guangdong Pugua	Hangzhou gourd	USVL1	-
Scaffolds Number	11	27	438	305 112
Scaffolds N50 (bp)	28 545 436	28 385 010	8 701 157	782
Contigs Number	11	71	18 083	365 668
Contigs N50 (bp)	28 545 436	11 208 824	28 343	537
Genome size (Mb)	298.7	297.3	313.4	176.7
Number of gaps	0	53	17 424	-
Length of gaps (Mb)	0	0.9	15.8	4.1
Centromeres annotated	11	-	-	-
Telomeres annotated	18	-	-	-
Number of gap-free chromosomes	11	0	0	0
Gene numbers	24 439	23 541	22 472	23 113
BUSCO (%)	97.5	95.5	95.4	-
LTR assembly index score	22.3	30.3	39.8	-
GC content (%)	35.01	32.0	31.5	32.0
Number of chromosomes	11	11	0	0
Assembly level	Chromosome	Chromosome	Scaffold	Scaffold
Assembly sequencing tech	PacBio Sequel	PacBio Sequel	Illumina HiSeq	Illumina HiSeq

a Bottle_gourd: https://www.ncbi.nlm.nih.gov/datasets/genome/GCA_000466325.1/

### Genome quality assessment

**Figure 1:**
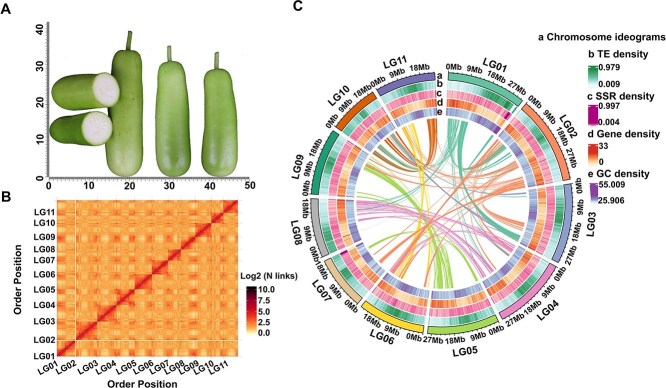
Pugua high-quality genome assembly. (A) The appearance of ripe fruit of Pugua. (B) Hi-C assembly result evaluation, darker colors indicate higher interaction frequency, and both the horizontal and vertical axes represent the order of each bin (300 000 bp) on the corresponding chromosome group. (C) Pugua genome information circle map. a, chromosome ideograms; b, TE density; c, SSR density; d, Gene density; e, GC density

From the perspective of genome assembly, the interaction intensity and location in the Hi-C assembled chromosome interaction heat map demonstrated that the 11 chromosomes were well assembled (Fig. 1B). The second-generation reads showed a quality of 99.25% and coverage of 99.97%, the third-generation CCS reads had a quality of 99.87% and coverage of 99.99%, and the third-generation ONT reads showed a quality of 95.96% and coverage of 99.99% (Table S2). These results further confirm the integrity of the assembled genome and the uniformity of sequencing coverage [20]. From a genetic perspective, the evaluation completeness of Benchmarking Universal Single-Copy Orthologs (BUSCO) based on OrthoDB10’s embryophyta database was 97.46% (Table S3). Furthermore, the genomic consistency quality value was 51.58, and the Long-Terminal Repeat Assembly Index value was 11.26, indicating that our assembled Pugua genome is of high integrity and accuracy.

### Genome annotation

We performed repeat sequence annotation of the assembled genome using de novo prediction results and a homologous species database. In total, we identified 114.90 Mb of transposable element (TE) sequences, accounting for 38.47% of the entire Pugua genome. Notably, retroelements comprised 24.79%, with long terminal repeat transposons (LTRs) making up 21.92% (Table S4). Tandem repeat sequences accounted for about 13.03 Mb or 4.36% (Table S4). The number of predicted genes and their genetic characteristics were similar to those of related species. Using GeMoMa [21] (v1.7), homologous species prediction indicated that these genes were primarily from other Cucurbitaceae species, including *B. hispida* (23 696 genes), *C. sativus* (22 172 genes), *L. siceraria HZ* (25 362 genes), and *L. siceraria Ls* (LLs) (20 865 genes). Statistical analysis using EVM [22] (v1.1.1) ultimately identified 24 439 coding genes, with an average gene length of 4199.64 bp and exon length of 1591.10 bp for each gene **(**Table S5). The number of predicted genes and genetic characteristics was consistent with those of the related species mentioned above. The completeness of gene prediction was evaluated using BUSCO, revealing that 96.34% of BUSCO genes were present in the Pugua genome, while 2.17% were incomplete and 1.49% were unpredicted, indicating high reliability and accuracy in gene prediction. Additionally, 1026 tRNAs, 3773 rRNAs, 87 miRNAs, and 28 pseudogenes were predicted and annotated. The predicted gene sequences were annotated using seven public databases, with 89.5% of the genes being annotated in at least one database (Table S6). Cellular anatomical entity genes accounted for the highest proportion in GO annotation classification statistics (Fig. S1B). KEGG enrichment analysis showed that these predicted genes were mainly involved in the MAPK signaling pathway—plant, plant hormone signal transduction, plant–pathogen interaction, starch and sucrose metabolism, phenylpropanoid biosynthesis, and biosynthesis of amino acids.

### Identification of telomere and centromere

Using the information on gene density, repeat sequence density, GC content, and collinearity, we mapped the circos diagram of the Pugua genome (Fig. 1C). Collinearity analysis, compared to the previous LLs genome version, showed structural rearrangements in the chromosomes (Fig. S2A), explaining our assembly’s achievement of 0 gaps. Only four telomere positions were not detected, namely the upstream telomeres of LG01 and LG07 chromosomes as well as the downstream telomeres of LG08 and LG10 chromosomes (Table S7). The remaining seven chromosomes were assembled in a gap-free and T2T manner (Fig. S2B). Among them, the longest telomere (12 960 bp) was located downstream of LG03 and the shortest one (1159 bp) downstream of LG09. The centromere lengths ranged from 484 028 to 5 429 881 bp (Table S8).

### Genomic evolutionary analysis

**Figure 2:**
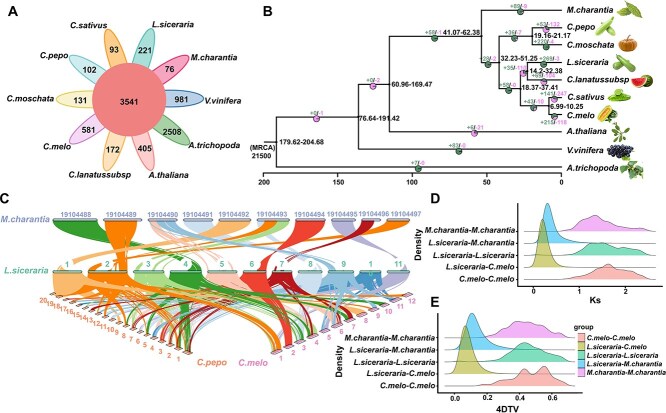
Evolutionary analysis of Pugua genome. (A) Clustering of gene families of various species The middle circle represents the number of gene families shared by all species, while the edges indicate the number of gene families unique to each species. (B) Evolutionary tree analysis, where the time on the evolutionary tree represents the divergence time supported by 95% of highest posterior density. The ‘+’ represents the number of gene families expanding on the node, the ‘-’ represents the number of gene families contracting on the node, and the pie chart represents the proportion of corresponding branch contraction and expansion gene families. The standard for significant expansion or contraction is that the family-wide *P*-values and Viterbi *P*-values are both less than .05. (C) Analysis of collinearity between the Pugua genome and closely related species genomes. (D) Distribution diagram of Ks values. (E) The peak of the 4DTv distribution map represents species divergence in the inter-species combination map, while the peak generated by the intra-species group cooperation map represents the whole genome replication event of the species

To obtain the phylogenetic position of Pugua, we conducted comparative genomic analysis using the genomic sequences of nine species and the genomic sequence of Pugua. Figure 2A shows that there are 3541 gene families shared among the selected species, and Pugua has 221 unique gene families (Table S9). Moreover, a phylogenetic tree with *A. trichopoda* as an out-group was constructed based on 1343 single-copy gene sequences (Fig. S3A, Fig. 2B). It was found that the divergence time between *A. thaliana* and Cucurbitaceae occurred approximately 60.96–169.47 million years ago (Mya). Subsequently, Pugua diverged from *C. lanatussubsp* approximately 14.3–32.38 Mya. We predicted 269 expanded gene families and 3 contracted gene families in the Pugua genome, with highly significant differences (Table S10). The Biological Process of GO enrichment analysis indicates that the genes of the expanded families are mainly involved in the carbohydrate metabolic process, rejection of self-pollen, cell wall organization, and oxylipin biosynthetic process (Fig. S3B), while the contracted gene families are involved in the lipid catabolic process (Fig. S3C).

### Genome-wide replication and collinearity analysis

Collinearity analysis was used to characterize the homogeneity of the Pugua genome and its related species (*Cucumis melo*, *Cucurbita pepo*, and *Momordica charantia*) (Fig. 2C). The lowest collinearity was observed with *C. melo* (58.61%), while the highest was with *C. pepo* (70.77%). These results indicate that chromosomal variation increases with the divergence of interspecies relations, promoting internal differentiation within Cucurbitaceae [23]. Whole genome duplication (WGD) events have played a key role in the origin and evolution of species. By analyzing the synonym substitution number (*Ks*) of each synonymous site, we found a landmark peak at *Ks* = 1.42 in the Pugua (Fig. 2D), indicating a single WGD event. This finding was confirmed by a four-fold synonymous (degenerative) third-codon transversion (4DTv) (Fig. 2E). The *Ks* distribution of Pugua homologous genes was compared with those of melon and bitter melon. The WGD event in the Pugua genome occurred before the divergence of Pugua and bitter melon and before the divergence of Pugua and melon (Fig. 2D and E). The sustained expansion of LTR retrotransposon (LTR-RT) insertion in the Pugua genome began at 0.92 Mya (Fig. S4), primarily involving Copia and Gypsy elements (Table S11), which underpin the genome expansion of Pugua.

### Metabolomic analysis

**Figure 3:**
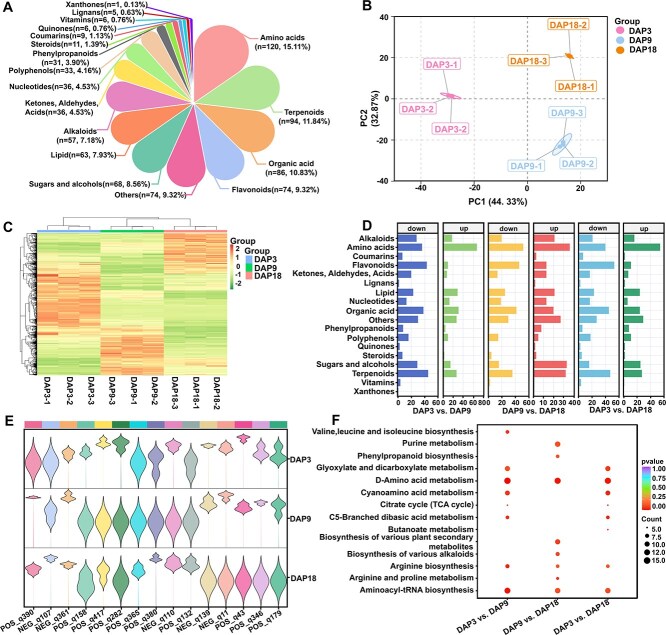
Metabolite analysis during Pugua development. (A) Proportion of metabolites (B) PCA analysis. (C) metabolite clustering heatmap. (D) The up and down-regulation distribution of metabolites in different comparison groups. (E) Violin plot of changes in metabolic substance abundance. POS_q390: tiglic acid; NEG_q107: citric acid monohydrate; NEG_q361: trans-caffeic acid; POS_q158: dihydrotestosterone; POS_q417: T-Glu-Phe; POS_q282: metformin; POS_q365: sinapyl alcohol; POS_q380: synephrine; NEG_q110: coniferaldehyde; POS_q132: coclaurine-1; NEG_q139: Dl-3-phenyllactic acid; NEG_q11: 2,3-dihydroxy-3-methylbutanoic acid; POS_q43: 3′-adenylic acid; POS_q346: quinidine; POS_q179: epibrassinolide. (F) KEGG enrichment analysis of metabolites

Using the LC-QTRAP platform, we qualitatively and quantitatively analyzed the metabolome of nine Pugua samples (DAP3, DAP9, and DAP18). We characterized and analyzed the changes in metabolites during different growth stages of Pugua fruit, identifying a total of 794 metabolites. These metabolites fell into 18 categories, including amino acids (120), terpenoids (94), organic acids (86), flavonoids (74), sugars and alcohols (68), etc. The results indicated that amino acids, terpenoids, sugars, and alcohols were the main metabolites involved in the growth and development of Pugua (Fig. 3A). Principal component analysis (PCA) clearly showed internal aggregation within each group of Pugua samples, with distinct separation trends at different growth stages, indicating significant differences in the composition of metabolic substances at DAP3, DAP9, and DAP18 (Fig. 3B). The clustering heat map revealed that the nine samples could be divided into three main clusters according to metabolite abundance changes, showing good repeatability among parallel clusters. Metabolite accumulation in DAP3 significantly differed from DAP9 and DAP18, with DAP9 and DAP18 exhibiting more similar accumulation patterns (Fig. 3C).

During the rapid expansion stage of Pugua fruit (DAP3 vs. DAP9), 620 differentially accumulated metabolites (DAMs) were identified, with 43.06% upregulated and 56.94% downregulated. In this period, terpenoid and flavonoid accumulation decreased, while amino acids and lipids increased (Fig. 3D). Changes in organic acid content were also notable, with tiglic acid (1270228.98-fold), citric acid monohydrate (861677.47-fold), and caffeic acid (615649.28-fold) showing the largest increases in abundance. Conversely, dihydrotestosterone (1.36E-05-fold), T-Glu-Phe (1.63E-05-fold), and metformin (3.39E-05-fold) showed the largest decreases (Fig. 3E). KEGG enrichment analysis indicated that metabolic processes at this stage mainly promoted protein synthesis, energy acquisition, and complex carbohydrate production, with decreased bitterness and increased umami taste (Fig. 3F)**.** During the ripening stage of bottle gourd fruit (DAP9 vs. DAP18), the abundance changes of 582 DAMs were similar to the rapid expansion period, with 43.47% upregulated and 56.53% downregulated. In this period, amino acids, flavonoids, and organic acids decreased, while sugars, alcohols, and terpenoids increased (Fig. 3D). Specifically, the accumulation of sinapyl alcohol (49278.77-fold), synephrine (49076.11-fold), coniferaldehyde (44161.16-fold), T-Glu-Phe (34416.78-fold), and coclaurine-1 (10695.77-fold) increased, while Dl-3-phenyllactic acid (4.46E-06-fold), 2,3-dihydroxy-3-methylbutanoic acid (5.78E-06-fold), 3′-adenylic acid (2.96E-05-fold), quinidine (4.42E-05-fold), and epibrassinolide (4.97E-05-fold) decreased (Fig. 3E). KEGG enrichment analysis showed that the main metabolic process was secondary metabolism, resulting in decreased umami flavor and increased carbohydrate content (Fig. 3F).

### Combined transcriptome and metabolism analysis

**Figure 4:**
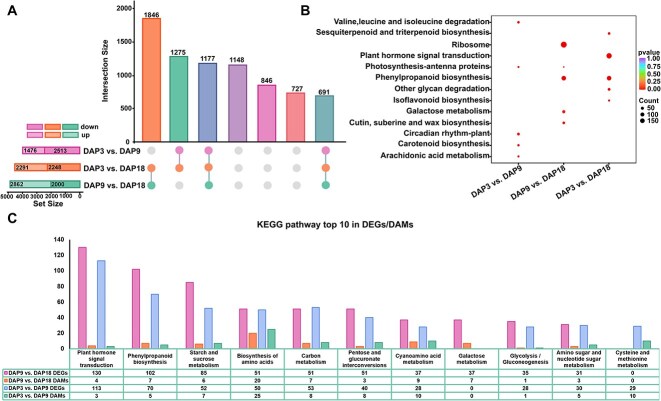
Combined analysis of transcriptome and metabolome. (A) UpsetR plot of DEGs distribution. (B) Differential gene KEGG enrichment analysis. (C) Metabolome and transcriptome combined KEGG enrichment analysis

During the rapid fruit enlargement stage (DAP3 vs. DAP9), 3989 differentially expressed genes (DEGs) were identified, with 1476 upregulated and 2513 downregulated (Fig. 4A). KEGG enrichment analysis showed that these DEGs were involved in photosynthesis—antenna proteins, arachidonic acid metabolism, and valine, leucine, and isoleucine degradation, encompassing photosynthesis, cell wall formation, and amino acid metabolism (Fig. 4B). During the fruit ripening stage (DAP9 vs. DAP18), 4862 DEGs were identified, with 2862 upregulated and 2000 downregulated (Fig. 4A). KEGG enrichment analysis indicated that these DEGs participated in the ribosome, phenylpropanoid biosynthesis, photosynthesis—antenna proteins, and cutin, suberine, and wax biosynthesis, involving protein synthesis, photosynthesis, and cell wall formation (Fig. 4B). Subsequent KEGG analysis of transcriptomes and metabolites showed that DAP3 vs. DAP9 and DAP9 vs. DAP18 were co-enriched in plant hormone signal transduction, phenylpropanoid biosynthesis, starch and sucrose metabolism, and amino acid biosynthesis pathways (Fig. 4C). These data indicated significant differences in photosynthesis, cell wall formation, amino acid metabolism, and carbohydrate synthesis at different developmental stages.

### Plant hormones synergistically regulate the fruit development of Pugua

**Figure 5:**
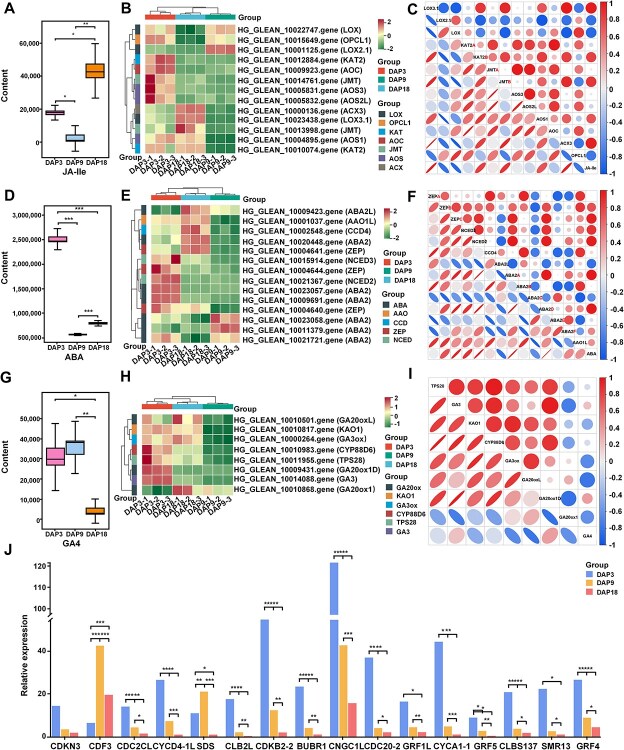
Regulation of plant hormone effects during the development of Pugua fruit. (A) JA IIe abundance changes. (B) Gene expression of jasmonic acid biosynthesis pathway. (C) Correlation heatmap between genes involved in the jasmonic acid biosynthesis pathway and metabolites. (D) Changes in the abundance of ABA. (E) Gene expression of ABA biosynthesis pathway. (F) Correlation heatmap between genes involved in ABA biosynthesis pathway and metabolites. (G) Changes in GA4 abundance. (H) Gene expression of gibberellin biosynthesis pathway. (I) Heat map of the correlation between gibberellin biosynthesis pathway genes and metabolites. (J) Regulating the expression of DEGs that regulate cell division and cell cycle. The digital markers after the genes in the heatmap are only used to distinguish different genes. The correlation heatmap calculation method is Pearson. The significance marker is the significant difference in the independent sample *t*-test, where ‘*’ represents *P* < 0.05, ‘**’ represents *P* < 0.01, and ‘***’ represents *P* < 0.001. CDF3: cyclic dof factor 3; SDS: cyclin-SDS isoform X2; CLB2L: G2/mitotic-specific cyclin-2-like; CNGC1L: cyclic nucleotide-gated ion channel 1-like isoform X2; GRF1L: growth-regulating factor 1-like; GRF5: growth-regulating factor 5 isoform X1; GRF4: growth-regulating factor 4

Fruit growth and development can be divided into several stages, with each stage transition regulated by different plant hormones [24]. We found that abscisic acid (ABA), and N-((−)-jasmonoyl)-S-isoleucine (JA-Ile) had their lowest abundance in DAP9. Notably, JA-L-Ile reached its highest abundance in DAP18, while the other hormones were most abundant in DAP3 (Fig. 5A and D). The abundance of gibberellin A4 (GA4) continuously decreased during development, showing no significant difference between DAP3 and DAP9 (Fig. 5G). The expression patterns of DEGs associated with jasmonic acid and abscisic acid synthesis aligned with the changes in JA-Ile and ABA abundance (Fig. 5A-F). However, DEGs associated with gibberellin synthesis showed an opposite pattern to the abundance changes of GA4 across different developmental stages (Fig. 5G-I), negatively regulating GA4 accumulation. In terms of plant hormone signal transduction, most DEGs transcripts were down-regulated in DAP3 vs. DAP9, including cyclin D3.2 protein (*CYCD3–1*), and SAUR family proteins (*SAUR50L* and *SAUR71L*). Conversely, transport inhibitor response 1 (*TIR1*), xyloglucan: xyloglucosyl transferase TCH4 (*TCH4*), auxin-induced proteins (*IAA14*/*IAA22DL*/*IAA29DL*/*AUX22L*), and indole-3-acetic acid-amido synthetase GH3.6 (*GH3.6*), which are involved in auxin signal transduction, were up-regulated. In DAP9 vs. DAP18, the transcripts of most DEGs showed an increase, including *SAUR21L/ SAUR50L/ SAUR71L* and *TCH4*, but *IAA14/ AUX22DL* and *CYCD3–1* were still down-regulated (Fig. S5A-E). Additionally, the expression of some DEGs that regulate cell division and the cell cycle decreased with the development of Pugua (Fig. 5J). These genes included cell division cycle 20.2 (*CDC20–2*), cell division control protein 2 homolog C-like (*CDC2CL*), cyclin-A1–1 (*CYCA1–1*), cyclin-D4–1 like (*CYCD4–1 L*), cyclin-dependent kinase B2–2 (*CDKB2–2*), cyclin-dependent protein kinase inhibitor SMR13 (*SMR13*), cyclin-dependent kinase inhibitor 3 (*CDKN3*), mitotic spindle checkpoint protein BUBR1 (*BUBR1*), and G2/mitotic-specific cyclin S13–7 (*CLBS137*). These findings enhance our understanding of the regulatory patterns across different developmental stages of bottle gourd, highlighting the complex interplay of plant hormones in fruit development.

### Cell wall relaxation remodeling adapts to Pugua development

**Figure 6:**
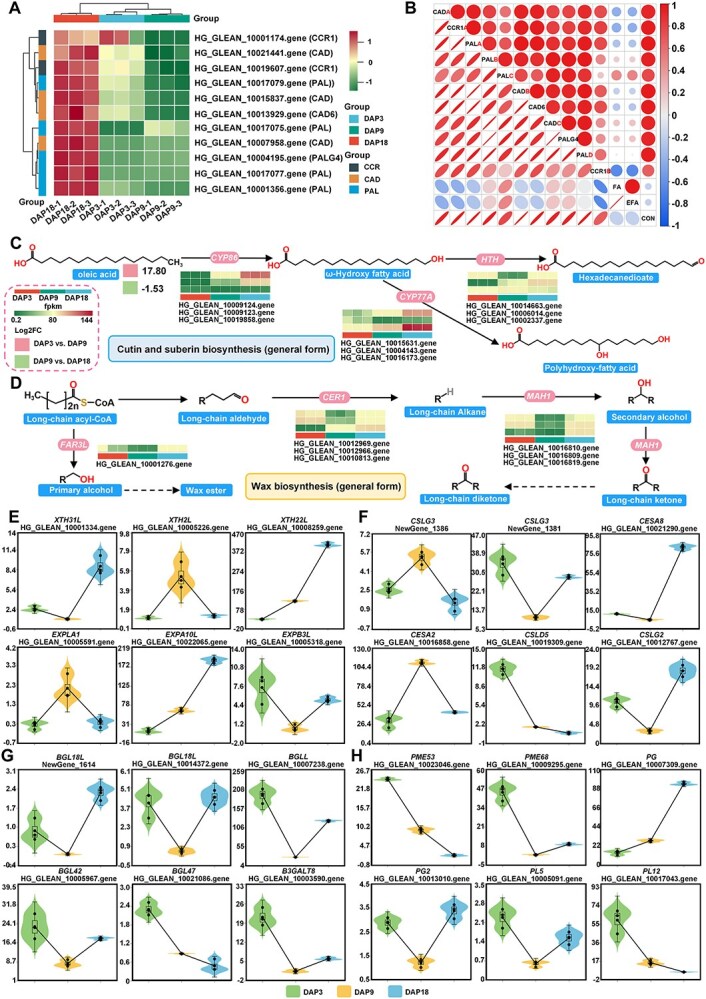
Regulation of cell wall remodeling during the development of Pugua fruit. (A) The biosynthetic pathway of phenylpropanoid. (B) Correlation heatmap between phenylpropanoid biosynthesis pathway genes and metabolites. The digital markers after the genes in the heatmap are only used only for distinguishing different genes. The calculation method for the correlation heatmap is based on Pearson. (C) Cutin and suberin biosynthesis. (D)Wax biosynthesis. (E) Gene expression of XTH and EXP families. (F) Expression of cellulose synthesis genes. (G) Expression of genes involved in pectin degradation. CON: coniferaldehyde; FA: Ferulic Acid; EFA: (E)-Ferulic Acid; XTH31L: xyloglucan endotransglucosylase/hydrolase protein 31-like; XTH2L: xyloglucan endotransglucosylase/hydrolase 2-like; XTH22L: xyloglucan endotransglucosylase/hydrolase protein 22-like; EXPLA1: expansin-like A1; EXPB3L: expansin-B3-like; EXPA10L: expansin-A10-like; CSLG3: cellulose synthase-like protein G3; CESA8: cellulose synthase A catalytic subunit 8; PME68: pectinesterase 68; PME53: pectinesterase 53 isoform X2; PG: polygalacturonase; PG2: pectinesterase 2; PL5: pectate lyase 5; PL12: pectate lyase 12; BGL18L: beta-glucosidase 18-like; BGLL: beta-galactosidase-like; BGL42: beta-glucosidase 42; BGL47: beta-glucosidase 47; B3GALT8: beta-1,3-galactosyltransferase 8

The cell wall of fruits and vegetables supports tissue morphology, and the relaxation and reconstruction of the cell wall, along with the formation of cutin and wax, change at different stages of fruit development to adapt to fruit expansion and ripening [25]. In DAP3 vs. DAP9, the abundance of phenylpropanoids was mainly down-regulated, with coniferaldehyde decreasing by 7.02E-05-fold. Similarly, the expression of cinnamyl-alcohol dehydrogenase (*CAD*) and cinnamoyl-CoA reductase 1 (*CCR1*), which encode rate-limiting enzymes for lignin synthesis, also decreased (Fig. 6A and B). In contrast, in DAP9 vs. DAP18, the abundance of phenylpropanoids was significantly up-regulated, with coniferaldehyde increasing by 44161.16-fold. Increased transcripts of phenylalanine ammonia-lyase (*PAL*), *CCR*, and *CAD* likely explain the rise in phenylalanine abundance during this stage. Cutin maintains fruit integrity and provides mechanical support [26]. Oleic acid, a crucial precursor for cutin and lignin synthesis, increased by 223487.61-fold in DAP3 vs. DAP9 but decreased by 0.35-fold in DAP9 vs. DAP18. In terms of DEGs in DAP3 vs. DAP9, genes involved in cutin biosyntheses, such as fatty acid omega-hydroxylase (*CYP86A7*)*,* protein HOTHEAD*-*like *(HTHL)*, and omega-hydroxypalmitate O-feruloyl transferase (*HHT1*), were up-regulated. Conversely, genes involved in the wax synthesis, including alkane hydroxylase MAH1 *(MAH1)*, very-long-chain aldehyde decarbonylase CER1 (*CER1*), and fatty acyl-CoA reductase 3-like (*FAR3L*), were down-regulated (Fig. 6C and D). In DAP9 vs. DAP18, the genes involved in both cutin and wax synthesis were mainly up-regulated, including *CYP77A1/CYP86A2/CYP86A7, CER1,* and *MAH1* (Fig. 6C and D). These results indicate that cutin accumulation is synchronized with the expansion and ripening of Pugua, while wax synthesis is limited during the rapid expansion stage. Additionally, we observed numerous dilation factors (XYH family and EXP family) and DEGs involved in cellulose synthesis and pectin degradation (Fig. 6E-H). Cellulose synthesis was predominant in DAP3 vs. DAP9, with pectin degradation genes being down-regulated. However, in DAP9 vs. DAP18, both cellulose hydrolysis genes and pectin-degrading genes were up-regulated. These findings suggest that the regulation of cell wall components is dynamic and stage-specific, contributing to the structural changes necessary for the growth and maturation of bottle gourd fruit.

### Dynamic regulation of resistance to developmental stress of Pugua

**Figure 7:**
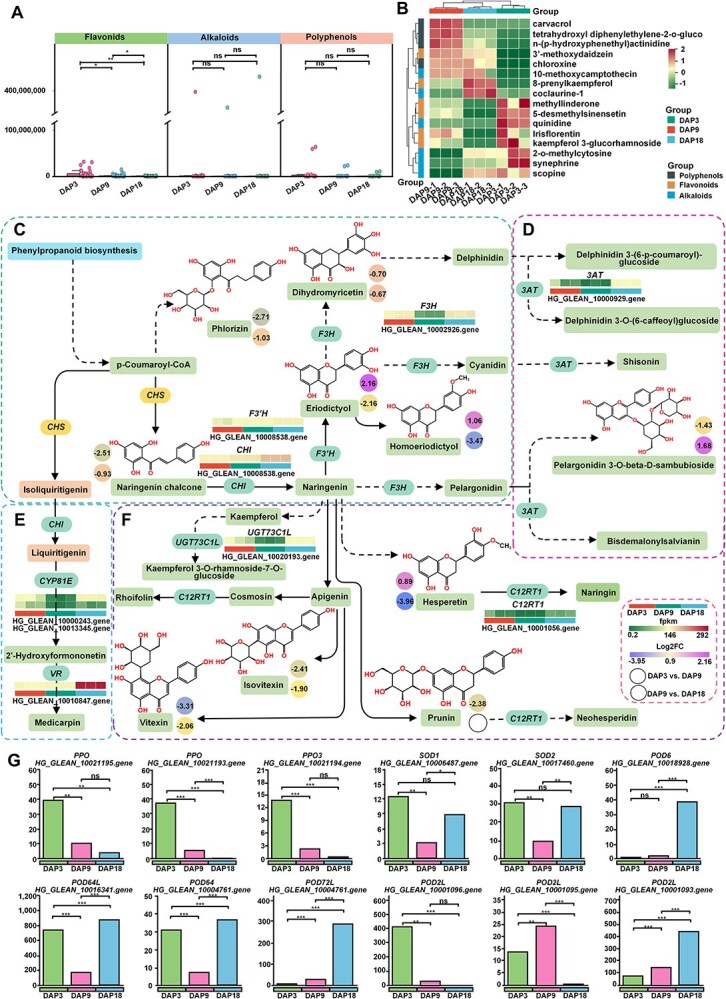
Dynamic regulation of stress resistance during the development of Pugua fruit. (A) Changes in overall abundance of flavonoids, alkaloids, and phenylpropanoids during fruit development. (B) Heatmap of changes in antioxidant abundance. (C) Flavonoid biosynthesis. (D)Anthocyanin biosynthesis. (E) Isoflavonoid biosynthesis. (F) Expression of antioxidant genes in flavone and flavonol biosynthesis. (G) Expression of antioxidant genes. The significance marker is the significant difference in the independent sample *t*-test, where ‘*’ represents *P* < 0.05, ‘**’ represents *P* < 0.01, and ‘***’ represents *P* < 0.001. CYP81E8L: cytochrome P450 81E8-like; UGT73C1L: UDP-glycosyltransferase 73C1-like; CHI: chalcone isomerase; CYP81Q32: cytochrome P450 81Q32; VRL: vestitone reductase-like; F3’H: flavonoid 3′-monooxygenase; F3H: naringenin 3-dioxygenase; C12RT1: flavanone 7-O-glucoside 2″-O-beta-L-rhamnosyltransferase; 3AT: anthocyanidin 3-O-glucoside 6″-O-acyltransferase; PPO: polyphenol oxidase; PPO3: polyphenol oxidase 3; POD6: peroxidase 6; POD72L: peroxidase 72-like; POD64L: peroxidase 64-like; POD64: peroxidase 64; POD2L: peroxidase 2-like; SOD1: superoxide dismutase 1; SOD2: superoxide dismutase 2

Cucurbitaceae plants are important sources of antioxidants [13]. Our metabolome results indicated that the abundance of most polyphenols, flavonoids, and alkaloids changed little and generally decreased as the Pugua developed (Fig. 7A). Further analysis of antioxidant substances that showed differences in all three stages revealed that only flavonoids showed differences during development. Therefore, the abundance changes of flavonoids during development are crucial for the antioxidant capacity of Pugua. However, the antioxidant substances that differ only at a certain developmental stage also determine the antioxidant capacity of the fruit at a specific developmental stage. The sudden increase in abundance of chloroxine (27869.58-fold), tetrahydroxyl diphenylethylene-2-O-gluco (20321.89-fold), N-(P-hydroxyphenethyl)actinidine (19689.62-fold), and carvacrol (17163.98-fold) in DAP3 vs. DAP9, enhancing the antioxidant capacity of Pugua at DAP9 (Fig. 7B). Similarly, the significant increase in synephrine (49076.11-fold), coclaurine-1 (10695.77-fold), scopine (9124.41-fold), and 2-O-methylcytosine (2814.56-fold) in DAP9 vs. DAP18 was related to the antioxidant capacity of Pugua at DAP18 (Fig. 7B). KEGG enrichment analysis revealed that these metabolites were primarily enriched in the flavonoid biosynthesis pathway. In DAP3 vs. DAP9, chalcone isomerase *(CHI)*, flavonoid 3′-monooxygenase *(F3’H),* and vestitone reductase-like *(VRL)* were up-regulated, while the transcripts of the other DEGs were decreased. In DAP9 vs. DAP18, the transcripts of most DEGs showed an increase (Fig. 7C-G). We also found that the transcript levels of polyphenol oxidase (*PPO/PPO3)* decreased as the fruit developed, while those of peroxidase *(POD6/POD64/POD64L/POD72L)* increased. Superoxide dismutase 2 *(SOD2)* and *SOD1* have the highest and lowest expression levels in DAP3 and DAP9, respectively (Fig. 7H). Additionally, genes encoding heat shock proteins (HSP), heat stress transcription factors, heat shock cognate proteins, cold-regulated proteins, universal stress proteins, WUSCHEL-related homeobox, and heavy metal-associated isoprenylated plant proteins (HIPP) were down-regulated in DAP3 vs. DAP9 (23 up-regulated and 24 down-regulated), while their expression was mainly up-regulated in DAP9 vs. DAP18 (31 up-regulated and 15 down-regulated) (Tables S12 and S13). The regulation of these resistance genes during the development of bottle gourd can inform the cultivation of high-resistance varieties.

### Amino acids, organic acids, and sugars regulate the formation of Pugua flavor

**Figure 8:**
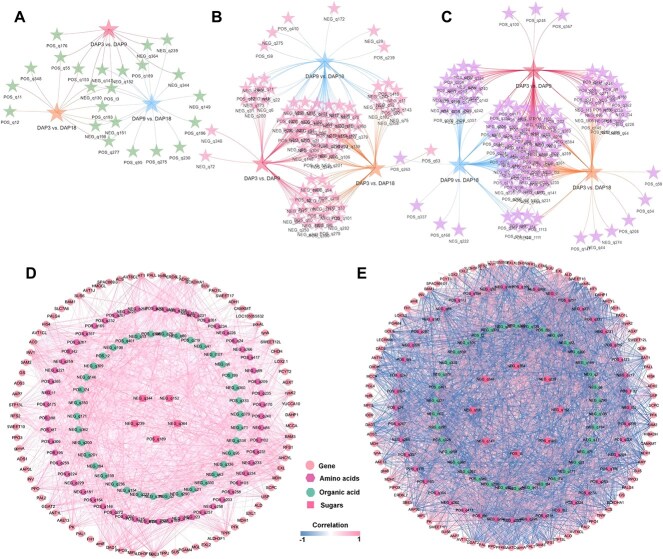
Flavor regulation during the development of Pugua fruit. (A) Differences in sugars at different stages. (B) Differences in organic acids at different stages. (C) Differences in amino acids at different stages. (D) Flavor regulation network in DAP3 vs. DAP9. (E) Flavor regulation network in DAP9 vs. DAP18

The overall metabolome analysis showed that amino acids, organic acids, and sugars are crucial components of Pugua flavor. The differences in these substances across different developmental stages are shown in Fig. 8A-C. In DAP3 vs. DAP9, the abundance of carbohydrates like sucrose, D-melibiose, and turanose mainly decreased, whereas in DAP9 vs. DAP18, their abundance mainly increased (Fig. 3D). The transcripts of beta-fructofuranosidase (*INV1/INV*), sucrose synthase 6 (*SUS6*), and sugar transport protein 13-like (*STP13L*) increased in DAP3 vs. DAP9, while the transcripts of *SUS*, sugar transport protein 14-like (*STP14L*), and bidirectional sugar transporters (*SWEET10/SWEET12L/SWEET16L/SWEET17*) decreased. In DAP9 vs. DAP18, the transcripts of sucrose-phosphate synthase (*SPS2/SPS4*), hexokinase-2 (*HK2*), *SUS/SUS5, STP13L/STP14, SWEET3b/SWEET7/SWEET10/SWEET12L, sucrose transport protein SUC4 (SUT4)*, and bidirectional sugar transporter N3 *(N3)* increased, while *SWEET7, SUS6*, and *HK3* were down-regulated. For amino acids, their abundance mainly increased in DAP3 vs. DAP9 but decreased in DAP9 vs. DAP18. In DAP3 vs. DAP9, DEGs in amino acid biosynthesis and metabolic pathways were mainly down-regulated, including glutamine synthetase (*GS*), aspartate aminotransferase (*AATC*), glutamate-glyoxylate aminotransferase 2 (*GGAT2*), and amino acid permease 3-like (*AAP3L*). In DAP9 vs. DAP18, the expression of DEGs is mainly up-regulated, including *GS, GGAT, AATC, AAP3L*, and amino-acid acetyltransferase NAGS2 (*NAGS2*). The expression of DEGs related to organic acid metabolism showed an opposite trend to that of amino acids, with up-regulated DEGs predominating in DAP3 vs. DAP9, including dihydrolipoyl dehydrogenase 2 (*DLD2*), malate dehydrogenase (*MDH1*), aconitate hydratase (*ACO*), and fumarate hydratase 1 (*FH1*). In DAP9 vs. DAP18, down-regulated DEGs predominated, including *MDH1, FH1, ACO,* and succinyl-CoA ligase [ADP-forming] subunit (*SUCLA1/SUCLA2*). To better understand the complex mechanisms of these flavor metabolites in the formation of Pugua flavor, we targeted the common DEMs in both comparison groups and selected DEGs with a correlation greater than |0.99| to construct a correlation network diagram of amino acid, organic acid, and glucose metabolism (Tables S14 and S15, Fig. 8D and E).

## Discussion

Bottle gourd is one of the oldest species of Cucurbitaceae cultivated by humans, prized for its soft texture, sweet taste, and medicinal properties, including diuretic and anti-cancer effects [27]. A complete and gap-free reference genome is the ultimate goal of high-quality genome assembly, as it aids in the conservation and innovation of bottle gourd germplasm resources [28]. Until now, the three published bottle gourd genomes have lacked completeness due to technical limitations [17–19]. In this study, we generated the first high-quality gap-free genome assembly of bottle gourd (Pugua), achieving a T2T complete assembly for seven chromosomes, with a genome size of 298.67 Mb. This is comparable to the genome sizes of bottle gourd ZAAS_Lsic_2.0 (297.90 Mb) [12] and slightly larger than zucchini (263 Mb) [29], but much smaller than the genome A36 of Chieh-qua (953.3 Mb) [30] and snake gourd (919.8 Mb) [31]. This may be because, on the evolutionary tree of Cucurbitaceae, Pugua is relatively close to zucchini in terms of branch position. After the evolutionary branching, Pugua experienced genomic expansion, accounting for 98.9% of the total expanded and contracted gene families [32]. In contrast, chieh-qua and snake gourd are relatively far from Pugua in terms of branch position, and their genomes have become large due to a large number of transposon replications during the evolutionary process [30, 31, 33]. *K-mer* analysis showed that the heterozygosity of Pugua was only .01%, much lower than that of chayote (.03%) [34], bitter melon (0.12%) [11], sponge gourd (0.60%) [35], and gynostemma pentaphyllum (0.90%) [12]. The low heterozygosity rate is the result of the homozygosity of genes caused by the self-fertilization process [36]. Our assembly represents the highest quality bottle gourd genome to date, providing valuable genetic resources for further studies on bottle gourd evolution and genetic improvement.

To further understand the origin of bottle gourd, we constructed an evolutionary tree and conducted a WGD event analysis. As previously reported, Pugua and watermelon form a sister clade with cucumber and melon [37]. Based on the *Ks* distribution of homologous genes between species, Wu *et al.* [18] estimated that bottle gourd and watermelon diverged about 10.4–14.6 Mya. Our results calculated that the divergence time between Pugua and watermelon is 14.3–32.38 Mya, and this time is closer to the divergence time of the Cucurbita genus at 30 ± 4 Mya [29]. The reason for the earlier and broader divergence period could potentially be that the prior genome sequencing depth and technology impacted the splicing of complete gene sequences in the comparative genomic analysis, thereby influencing the determination of the divergence time [38]. The results will contribute to a further understanding of the origin of Pugua and provide a higher resolution timeline for the evolution of Cucurbitaceae species. The Cucurbitaceae is predicted to have undergone four WGD events [37]. Analysis based on *Ks* distribution and 4DTV distribution indicates that there has been no recent WGD event in Pugua since the ancient tetraploidization event, which is consistent with the previous relevant research findings on watermelon [39], and bottle gourd [17]. It confirms the statement that Cucurbitaceae plants, apart from the core-eudicot common hexaploidization (ECH), have evaded more recent whole-genome duplication events [40]. The insertion time of LTR-RTs in Pugua is 0.92 Mya, indicating that its amplification occurred recently after species formation and may promote phenotypic diversification, providing data for the evolutionary study of Pugua [41]. After ancient triploidization [42], Pugua has chosen a stable gene family changing pattern, avoided recent WGD events, and adapted to the environment through the adjustment of specific gene families, which provides important information for understanding the evolutionary mechanisms and diversity of Cucurbitaceae.

### Plant hormones regulating Pugua fruit development

Plant hormones, as trace organic compounds produced in plants, have complex physiological effects and can significantly influence the entire process of fruit growth, maturation, and senescence through signal transduction [43]. In blueberry fruits, higher levels of GA, SA, JA, and ABA during early development, and higher ABA and lower GA levels at the mature stage, make the fruits of lateral racemes that bloom later grow and mature faster than those of terminal racemes that bloom earlier [44]. This is similar to the hormonal situation in Pugua, ABA, JA-Ile, and GA4 showed high abundance in DAP3, but only GA4 showed low abundance in DAP18. This pattern suggests that increased GA levels in early fruit development induce cell division and elongation, which is inhibited by decreased GA and increased ABA levels in the maturation stage [45]. Although auxin and GA are the primary hormones regulating cell division and expansion, auxin is crucial for cell enlargement in early fruit development [46]. In strawberry fruits, the genes of the Aux/IAA family (*FaAux/IAA26a, FaAux/IAA27a, FaAux/IAA27b*) are highly expressed in the early stage, and another study shows that the expression of auxin regulatory genes decreases during the strawberry maturation stage [47]. In the rapid expansion stage of Pugua, the genes of the Aux/IAA family (*IAA14/IAA22DL/IAA29DL/AUX22L*) are up-regulated, but in the mature stage, *IAA14/AUX22DL* are down-regulated, confirming their important regulatory role in growth and negative regulatory role in maturation [48]. Meanwhile, the expression levels of CDC genes and CYC genes (*CDC20–2/ CYCA1–1/ CYCD4–1 L, CDKB2–2/ CYCD3–1*) involved in cell cycle regulation in bottle gourd fruits decrease with development. Similar gene expression changes are also observed in blueberry fruits, where *VcCDKB2;2* and *VcCycD3;1 L* are highly expressed in the early stage but decline during maturity [49]. These gene expressions are highly coordinated with fruit development stages and physiological requirements. In the early stage, with cell division and proliferation as the main processes, high gene expression satisfies the initial requirements. In the later stage, decreased gene expression slows cell division and enables cells to enter the endoreduplication cycle to adapt to the needs of substance accumulation and quality formation [50, 51]. ABA and ethylene are considered the main plant hormones mediating the maturation of fruits. Although the ethylene synthesis genes are highly expressed in the mature stage of Pugua fruits, affected by the up-regulation of *CTR1, ERF1B* is down-regulated. At this time, the high content of ABA and the high expression of ABF family genes (*ABF3/ABI5/DPBF3*) indicate that ABA is the main plant hormone mediating the maturation of Pugua.

### Cell wall relaxation and remodeling in Pugua development

The final size and shape of the fruit are determined by cell division and cell elongation during fruit development. Wall relaxation and cell expansion are achieved by the dynamic modification of the cell wall, which is located in the outermost layer of the plant cell [52]. Specifically, in the early stages of fruit development, cells primarily rely on the plasticity of primary cell walls (composed of cellulose, hemicellulose, and pectin) to grow and expand. As the fruit matures, some cells stop growing and begin to form secondary cell walls (composed of cellulose, hemicellulose, and lignin) to provide mechanical support [53]. Cell wall relaxation factors such as EXP and XTH mediate cell wall relaxation during cell elongation, with EXP modifying the cellulose network to promote tensile stress for expansion and XTH cutting and reconnecting hemicellulose chains in the xyloglucan–cellulose network [54, 55]. For example, in mango, increasing the expression of EXP and XTH genes can accelerate the development and expansion rate of mango fruits [56]. During the rapid expansion stage of Pugua, the up-regulation of genes such as *XTH2L/XTH22L* and *EXPLA1/ EXPA10L* contributes to cell wall relaxation. In the mature stage, slow growth is mediated by the up-regulation of *XTH22l/XTH31L* and *EXPB3L/EXPA10L*. In loquat fruits, the regulation of EXP genes on fruit development is manifested as *EjEXP2* being only expressed during the rapid cell amplification period and the expression level of *EXP1* remaining stable throughout the development process [57]. The cuticle is a protective layer on the outer surface of the fruit cell wall, forming a waterproof barrier that maintains internal water balance and increases the smoothness of the fruit surface [58]. During the period from DAP3 to DAP9, the abundance of the common precursor oleic acid for cutin and suberin biosynthesis increases, and the expression levels of its downstream genes *HTHL, HHT1,* and *CYP86A7/CYP86A2* increase. However, during the period from DAP9 to DAP18, the abundance of oleic acid decreases, but the genes *CYP77A1/ CYP86A1/CYP86A2/CYP86A7/CYP94A5* for cutin, suberin, and wax biosynthesis are up-regulated. Similarly, in navel orange, the gene *CYP86A8* involved in cuticle formation is highly expressed in the early stage of fruit development, while the expressions of *CYP86A1* and *CYP86A2* increase in the later stage of development [25]. These results will be helpful for further studying the dynamic development of cell walls during the development of Pugua fruits.

### Antioxidant and stress resistance in Pugua development

Cucurbitaceae plants are rich in bioactive substances such as polyphenols, flavonoids, alkaloids, and cucurbitin. These phytochemicals benefit human health and help plants resist various stresses [59]. During Pugua development, the composition and content of polyphenols, flavonoids, and alkaloids dynamically change. Although most antioxidants experienced a slight decrease during development, the overall antioxidant capacity at specific stages is likely related to the sudden increase of antioxidant substances. For instance, the abundance of chloroxine, p-Hydroxyphenethyl actinidine, carvacrol, broussonin B, tetrahydroxyl, and diphenylethylene 2-O-glucose significantly increased from DAP3 to DAP9, enhancing the antioxidant capacity during this rapid expansion stage. Similarly, the significant increase of alkaloids such as synephrine, coclaurine-1, scopine, and 2-O-methylcytosine during DAP9 to DAP18 contributes to antioxidant capacity during maturation. These antioxidants protect cells from damage by neutralizing free radicals and reducing oxidative stress [60]. The expression of flavonoid synthesis-related genes was mainly down-regulated from DAP3 to DAP9 but up-regulated from DAP9 to DAP18. However, the expression level of *CHI* is increasing throughout the whole development process. In raspberries, the SOD activity is positively correlated with the expression level of SOD genes. *RuSOD1* and *RuSOD2* show a sharp increase only in S2 among the four developmental periods of yellow raspberry fruits and are hardly expressed in S4 [61]. In Pugua, *SOD1* and *SOD2* have the highest expression levels at DAP3 and the lowest at DAP9. This difference may stem from the differences in plant varieties themselves [62, 63]. Plants have evolved unique defense mechanisms to cope with various biotic and abiotic stresses, such as extreme heat, cold, heavy metals, and pathogen attacks [64]. Heat stress can cause protein dysfunction and aggregation, but HSPs act as molecular chaperones to prevent protein denaturation and promote the refolding of heat-damaged proteins, reducing toxic effects on plant cells [65]. The functional identification of the rice *OsHIPP42* mutant line indicates that the destruction of this gene will make rice sensitive to excessive copper (Cu) and zinc (Zn) [66]. Similarly, *CsHIPP36* is related to the tolerance of tea trees to cadmium (Cd), and HIPP5 and HIPP6 are related to the degradation of the endoplasmic reticulum in Arabidopsis [67, 68]. During the development of the Pugua fruit, from DAP3 to DAP9, *HIPP5/HIPP31/HIPP20L* are down-regulated and *HIPP28L/HIPP21L* are up-regulated; from DAP9 to DAP18, *HIPP3/HIPP5/HIPP6/HIPP21L/HIPP20L* are up-regulated and *HIPP36/HIPP39/HIPP42* are down-regulated, showing that the Pugua fruit can cope with stress by regulating the expression of specific genes at different developmental stages. In particular, HSP70s play an important role in the stress tolerance of various plants such as cabbage, tomato, and pepper, which further illustrates that there are multiple regulatory mechanisms when plants deal with stress [69].

### Flavor in Pugua development

The umami taste of bottle gourd fruit is peculiar, which is the complex result of the synthesis and metabolism of various amino acids, and free glutamic acid is the main umami component of bottle gourd [54]. We found that the abundance of L-glutamic acid decreased first and then increased during the development of the Pugua. In contrast, the abundance of L-aspartic acid, another recognized umami amino acid, increased first and then decreased. Correlation analysis showed that *PALG4* had a strong positive correlation with L-glutamic acid, and *AHCYL* had a strong positive correlation with L-aspartic acid. *GGAT2* can catalyze the reactions in the photorespiratory pathway and contribute to amino acid metabolism in the nitrogen cycle, including catalyzing relevant transamination and reverse reactions [70]. Nitrogen is incorporated into the C2 cycle through glyoxylate transamination, thereby affecting the metabolism and accumulation of glutamic acid [71]. Amino acid permeases (AAPs) genes are expressed in various tissues of plants and are involved in the loading of xylem and phloem, as well as the transportation and absorption of amino acids between source and sink [72]. During this process, AAPs preferentially transport major amino acids such as glutamic acid, glutamine, and asparagine based on the concentration and pH of amino acids in the apoplast [73]. During the ripening period of Pugua, the expression level of *AAP3L* (HG_GLEAN_100133055) increases, and this gene has been predicted as a key gene for the formation of bottle gourd flavor in the research by Wang *et al.* [27]. *AAP3* has been proven to specifically bind to basic amino acids, but over-expression of *OsAAP3* in rice can also increase the concentration of L-aspartic acid [74, 75]. Therefore, we speculate that during Pugua fruit development, *AAP3L* may be crucial for amino acid transportation and flavor formation, influencing the accumulation of umami-related amino acids like L-glutamic acid and L-aspartic acid. The formation of Pugua flavor is also affected by soluble sugar and organic acids, which interact with free amino acids to further enrich the flavor level of Pugua. The tricarboxylic acid cycle (TCA) is an important intermediate pathway involved in the biosynthesis of amino acids and organic acids [76]. The soluble sugars in fruit are mainly in the form of monosaccharides and partial disaccharides such as sucrose, glucose, and fructose [77]. The abundance of sucrose, D-melibiose, turanose, and maltose indicated that the sweetness of the fruit was mainly accumulated during the ripening stage after expansion. In a review of Cucurbitaceae, two genes involved in regulating the flavor of bottle gourd were summarized -phosphate synthase 4 (*SPS4*) and sucrose synthase 5 (*SUS5*) [37]. We found up-regulated expression of *SUS6* and *INV* from DAP3 to DAP9, and *SUS/SUS5* and *SPS2/SPS4* from DAP9 to DAP18. The SWEET family is plant transporters involved in the outflow of disaccharides or monosaccharides from high to low concentrations to exosomes [78]. *SWEET10* was differentially expressed in Pugua at different stages of fruit development and was consistent with the abundance of sucrose. This is similar to the involvement of *AcSWEET10* in saccharose accumulation in melons through high expression during the early ripening period [79]. These results provide useful information for the analysis of the flavor changes during the development of bottle gourd.

## Conclusion

**Figure 9:**
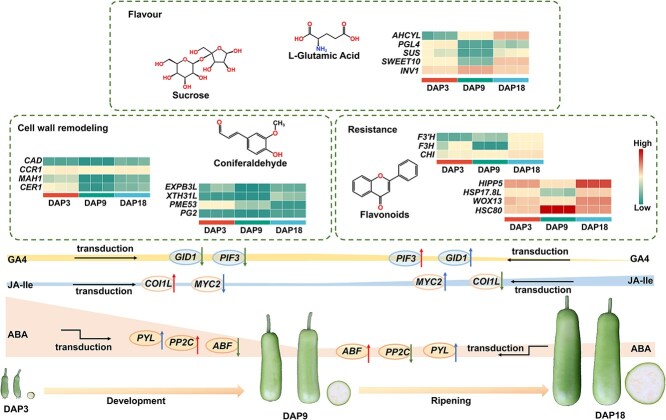
A network model was constructed based on transcriptome and metabolome to illustrate the potential regulatory mechanisms of Pugua fruit development. The direction of the arrows is used to indicate changes in gene expression, arrows pointing upwards represent an increase in expression levels, and arrows pointing downwards represent a decrease in expression levels.

In this study, we presented a gap-free T2T genome of bottle gourd (Pugua) and conducted thorough transcriptome and metabolome analyses across various developmental stages of Pugua fruit, shedding light on the dynamics of plant hormones, cell wall remodeling, stress resistance, and the regulation of flavor formation (Fig. 9). This work has solidified the theoretical foundation for understanding the regulatory mechanisms governing Pugua fruit maturation and quality metabolism. Overall, not only provides novel insights for evolutionary studies within the Cucurbitaceae family but also constitutes a valuable resource for the functional genomics of Pugua fruit. The focus on Pugua fruit development is particularly significant, holding substantial implications for future molecular breeding and quality control of Pugua, thus charting a course for enhanced cultivation practices and genetic enhancement.

## Materials and methods

### Collection of plant material

The Pugua (Guangdong Pugua) (*L. siceraria* (Mol.) Standl.) used in this study was a homozygous inbred line derived from the native variety ‘Huizhou bottle gourd’ in Guangdong. Pugua has early maturing characteristics and shows short cylindrical in shape, with a shiny green skin and without grooves for fruit. Seedlings at 10 days after sowing were collected for genomic DNA extraction and sequencing, and fruits of DAP3 (tender fruit), DAP9 (commercial fruit), and DAP18 (mature fruit) were collected for transcriptomic RNA extraction and sequencing. The sampling rule is to take the seeds from the middle of the fruit to preserve the skin and flesh, and mix the eight fruits evenly at each stage.

### Genome sequencing

Genomic DNA was extracted from fresh young leaves of Pugua by modified cetyltrimethylammonium bromide method in strict accordance with the manufacturer’s instructions [80]. Nanodrop 2000 spectrophotometer (Thermo Fisher Scientific, Wilmington, DE) and Qubit 3.0 Fluorometer (Life Technologies, Carlsbad, CA, USA) assessed extracted DNA concentration and purity, and 0.35% agarose gel was tested for integrity by pulsed-field electrophoresis. The short read library was constructed and sequenced in strict accordance with the standard protocol provided by Illumina (Illumina, San Diego, CA, USA). Genome long reading library construction and sequencing were performed according to the standard protocol provided by Oxford Nanopore Technologies Ltd (Oxford, UK). The SMRTbell Express Template Prep Kit 2.0 (Pacific Biosciences, USA) was used to perform the PacBio HiFi (CCS) database according to the standard protocol provided by PacBio. Sequencing was performed in the Sequel II system. For the Hi-C library construction scheme, fresh Pugua leaves tissue was cracked, and purified DNA was broken into 300–700 bp fragments for high-throughput sequencing using the Illumina platform (PE150).

### Genomic characterization assessment

Genome size, repeat ratio, and heterozygosity were evaluated using a *K* = 21 *K-mer* profile constructed from 350 bp short-read library data. *K-mer* counts are performed using jellyfish (2.1.4), with the parameter set to -h 10 000 000 000. The genome profile was calculated using genome scope (2.0) with the parameter set to -k 21-p 2-m 100 000. Using diploid as the fitting standard, the first peak corresponds to a *K-mer* depth of 22.70, and the genome size is calculated to be 297.40 Mb.

### Genome assembly and assembly evaluation

Using high-quality genome length data, the contig version of the genome was assembled using hifiasm (v0.19). The complete evaluation of the preliminary assembly of the genome draft was conducted by BUSCO (v5.2.2, https://busco-data.ezlab.org/v4/data/lineages/), BWA (V0.7.10), Minimap2 (2.24 - r1122), LTR_FINDER (v1.0.7), LTRharvest (v1.5.9), and LTR_retriever (v2.8) finish, accuracy by Merqury (1.3, GitHub: https://github.com/marbl/merqury). For chromosome mounting, BWA (v0.7.17) was used to compare the Hi-C data to the genome sequence at the contig level to filter out invalid data. Lachesis software was used to sequence and orientate the contig version genome by using the interactive signals generated by Hi-C data and finally mount it to the chromosome version. The Hi-C assembled chromosome genome is equally divided into a 300 000 bp bin. The number of Hi-C read pairs between any two bins is used as the intensity signal of interaction between the two bins and is displayed in heat map form by R language (https://www.r-project.org/). Circlize software was used to map gene density, repeat sequence density, GC content, and collinearity. Diamond (v0.9.29.130) was used to compare the gene sequences of different versions of bottle gourd, and the C-score value was filtered by JCVI (v0.9.13) software to identify similar gene pairs (e < 1e−5, C score > 0.5). The genes in the collinear block were obtained by MCScanX to verify the accuracy of genome assembly**.** The potential telomere repeat units and centromere repeats of Pugua genome were processed, respectively, using TIDK (https://github.com/tolkit/telomeric-identifier) and Centromics (https://github.com/ShuaiNIEgithub/Centromics) software. Location and sequence using FindTelomeres telomeres (https://github.com/JanaSperschneider/FindTelomeres) based on repeat unit. The location and sequence of the centromere can be obtained by back comparing the bottle gourd genome.

### Repeat sequence annotation

We adopted RepeatModeler2 (v2.0.1) for the automated genomic discovery of TE families mobilized RECON(v1.0.8) and RepeatScout(v1.0.6) for de novo prediction of the genome. LTR *de novo* prediction is specially performed by LTR_retriever(2.9.0), which can be used to integrate known databases to remove redundancy and obtain a specific repeat sequence database for bottle gourd. With the help of RepeatMasker (v4.1.2), the transposon sequence (TE) prediction is performed for the bottle gourd genome. Tandem repeats were predicted using the MIcroSAtellite identification tool (MISA v2.1) and Tandem Repeat Finder (TRF, v409).

### Coding gene prediction and annotation

The coding gene prediction methods are de novo prediction, homology prediction, and transcriptome prediction. We used Augustus (v3.1.0) and SNAP (2006-07-28) for genetic *de novo* prediction. Homologous species predictions were based on GeMoMa (v1.7) for four homologous species, *B. hispida, C. sativus, L. sicerariaHZ,* and *LLs*. Transcriptome gene prediction was performed by GeneMarkS-T (v5.1) and PASA (v2.4.1). The predicted genes of the bottle gourd genome were obtained by integrating the results from the above three methods with EvidenceModeler (v1.1.1) and PASA (v2.4.1), and then the completeness was assessed by BUSCO (v5.2.2). The annotation of pseudogenes was completed using GenBlastA (v1.0.4) and GeneWise (v2.4.1). Annotation analysis of predictive gene sequences was performed by NR, eggNOG, GO, KEGG, TrEMBL, KOG, SWISS-PROT, and Pfam.

### Genome evolution analysis

Orthofinder v2.4 software was used for family classification of the protein sequences of 10 selected species. The gene families obtained were annotated in the PANTHER (V15) database, and the bottle gourd-specific gene family was analyzed by clusterProfile (v3.4.4) for GO and KEGG enrichment. Using the method of Fu *et al.* [11], the evolutionary tree was constructed using the single copy genes of each species in the gene family. The divergence time was calculated using MCMCTREE of PAML (v4.9i) software. Furthermore, based on the evolutionary tree, CAFE (v4.2) was used to predict the contraction and expansion of the bottle gourd gene family relative to its ancestors. The significance of contraction or expansion was determined according to the criterion that both family-wide *P*-values and Viterbi *P*-values were less than .05. A forward selection analysis was then performed based on the CodeML module in PAML (v4.9i).

### WGD events and collinearity analysis

WGD event analysis adopts Ks and 4DTv methods to predict. *Ks* analysis of homologous genes in bottle gourd was performed using wgd (v1.1.1) software, 4DTV calculated, using the script https://github.com/JinfengChen/Scripts HKY alternative model correction. The method of collinearity analysis is consistent with previous collinearity analysis to verify the accuracy of genome assembly. LTR_FINDER (v1.07) and LTRharvest (v1.5.9) are still used to search for LTR-RT sequences, and LTR_retriever (v2.8) is used for consolidation filtering to obtain highly accurate LTR-RT. The LTR sequences on both sides were extracted using MAFFT (v7.205) for comparison, and the distance and insertion time were calculated using EMBOSS (v6.6.0) based on the Kimura model.

### Transcriptome sequencing and analysis

The total amount of three samples (three replicates per sample) was 1 μg. Use Hieff NGS Ultima Dual-mode mRNA Library Prep Kit for Illumina (Yeasen Biotechnology (Shanghai) Co., according to manufacturer’s instructions. Ltd.) generated a sequencing library and sequenced it on the Illumina NovaSeq platform to generate 150 bp double-terminal sequences. All raw readings are further processed on the bioinformatics analysis platform BMKCloud (www.biocloud.net). HISAT2 (2.0.4) and StringTie (v2.2.1) software were used to accurately compare and assemble clean reads with LagenariaHZ. Gene function annotation was based on NR, eggNOG, GO, KEGG, TrEMBL, KOG, SWISS-PROT, and Pfam by sequence alignment. DESeq2 (1.30.1) software was used for differential expression analysis, and the screening criteria were Fold Change ≥2 and false discovery rate < .01.

### Metabolite extraction and analysis

After freeze-drying the bottle gourd fruit, the superliquid was extracted by adding the extraction solution, magnetic bead grinding, ultrasonic treatment, static centrifugation, and other steps in the UPLC-ESI-MS /MS system (UPLC, Waters Acquity I-Class PLUS; MS, Applied Biosystems QTRAP 6500+) detection analysis. The multiple reaction monitoring model of triple quadrupole mass spectrometry was used for qualitative and quantitative analyses of metabolic substances based on the self-built database GB-PLANT. Metabolites were annotated by KEGG, Human Metabolome Database, and Lipid Metabolites and Pathways Strategy. Variable importance in projection (VIP), *P*-value, and fold change were combined in metabolic difference analysis. The screening criteria were fold change ≥1, VIP ≥ 1, and *P-*value <.05.

### Combined transcriptome and metabolome analysis

Based on the analysis results of DEGs and DAMs, DEGs and DAMs of the same group were mapped to the KEGG pathway simultaneously. Significant enrichment was determined by Fisher’s exact test, and the standard was *P*-value (*p*_bonferroni) ≤ .05.

## Supplementary Material

Web_Material_uhae335

## Data Availability

The *L. siceraria* (Molina) Standl (Pugua) raw genome sequencing, assembly data and TE notes are available from the NCBI under project ID PRJNA1196720.
