# Supplementary material for: Combined genomic, transcriptomic, and metabolomic analyses provide insights into the fruit development of bottle gourd (Lagenaria siceraria)
Source: Hortic Res. 2024 Nov 27;12(3):uhae335. doi: 10.1093/hr/uhae335 (PMC11883228; doi:10.1093/hr/uhae335)
Supplement: Web_Material_uhae335 [file web_material_uhae335.zip › Supplemental Figures.docx]

Supplemental Information


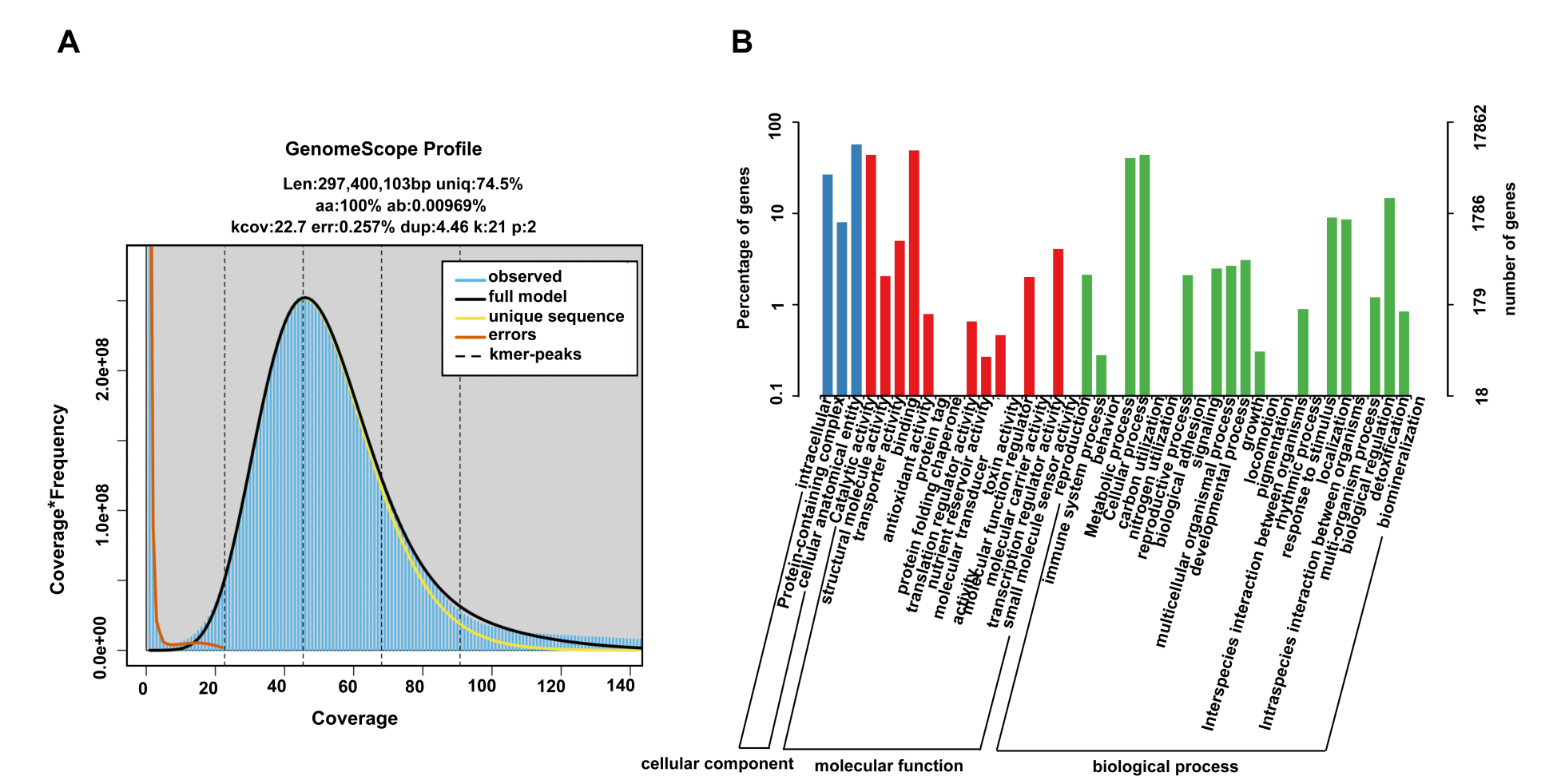


Figure S1 Genomic karyotype analysis and prediction of gene function annotation. (A) Kmer distribution. (B) prediction of gene enrichment for each secondary function of GO. The horizontal axis represents the content of each category in GO, the left side of the vertical axis represents the percentage of genes, and the right side represents the number of genes.


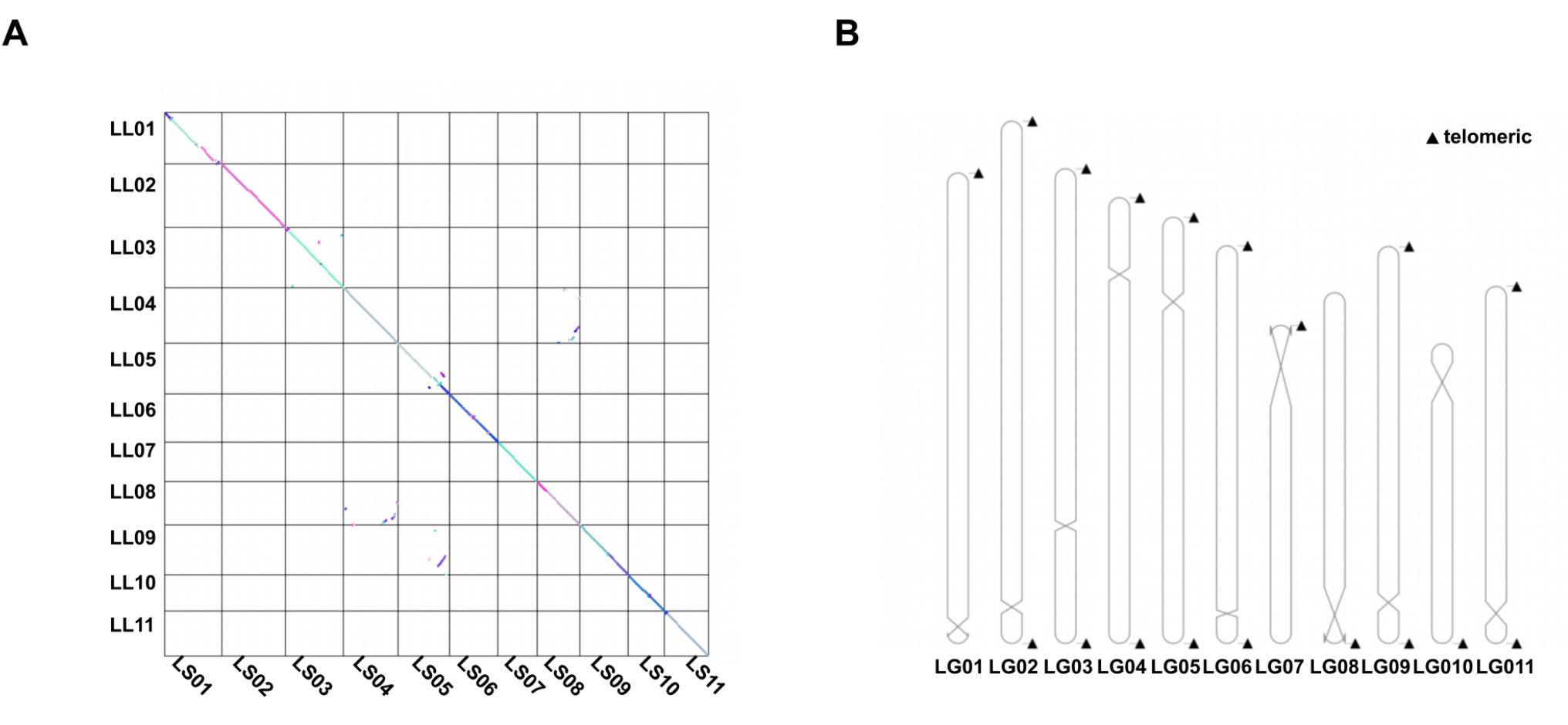


Figure S2 The assembly of the Pugua genome. (A) Involves collinearity analysis between the Pugua genome and the previous version of the genome. Filtering out non major corresponding chromosome collinearity genes with less than 100 genes (intra species chromosome collinearity is not filtered). (B) Pugua genome chromosomes.


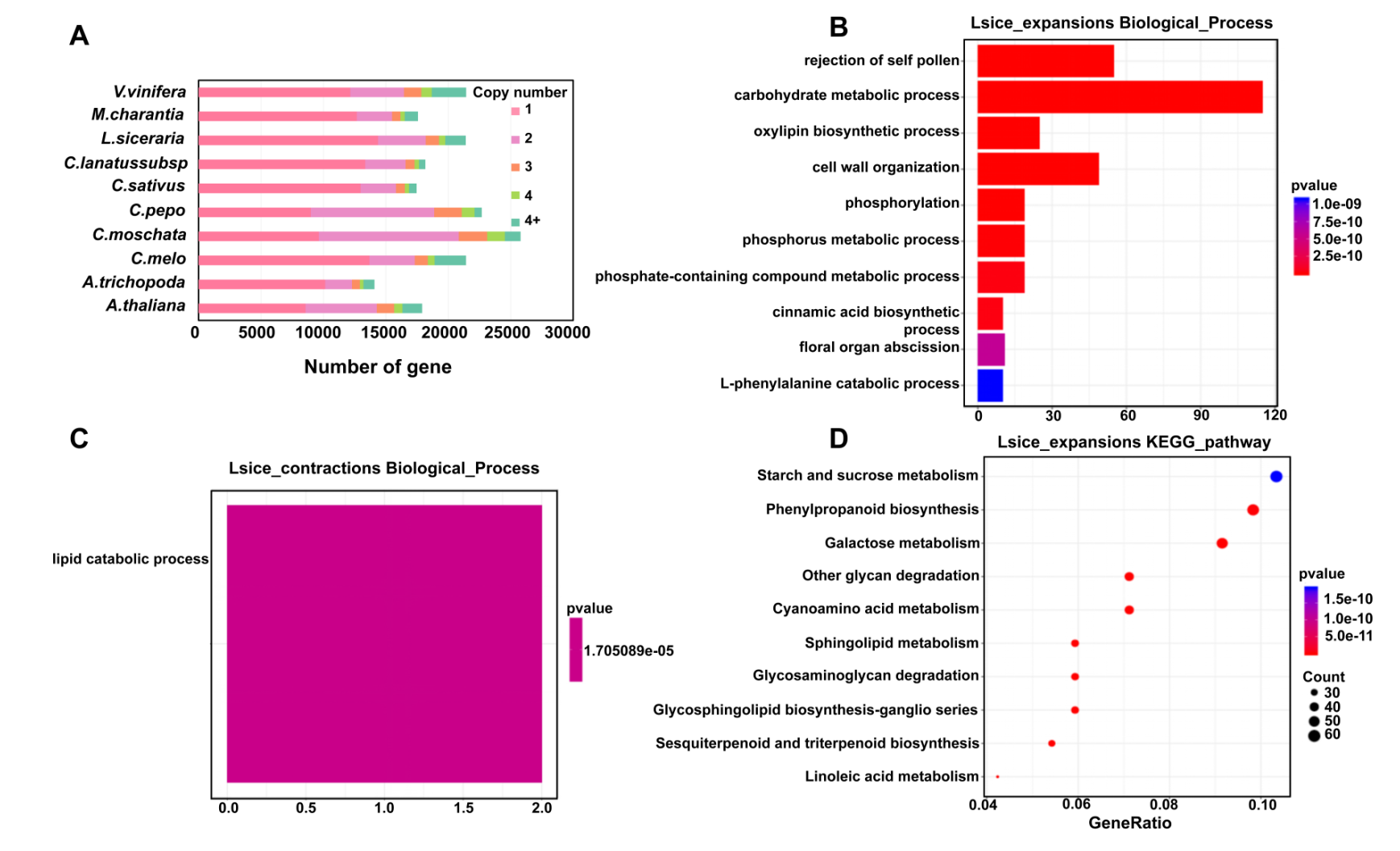


Figure S3 Distribution of single copy genes in various species' gene families and gene family functional annotation. (A) Distribution of single copy genes in all gene families for each species. (B) Pugua expansion gene family GO enrichment analysis. (C) Gene family GO enrichment analysis of contraction genes. (D) Enrichment analysis of expanded gene family KEGG enrichment analysis.


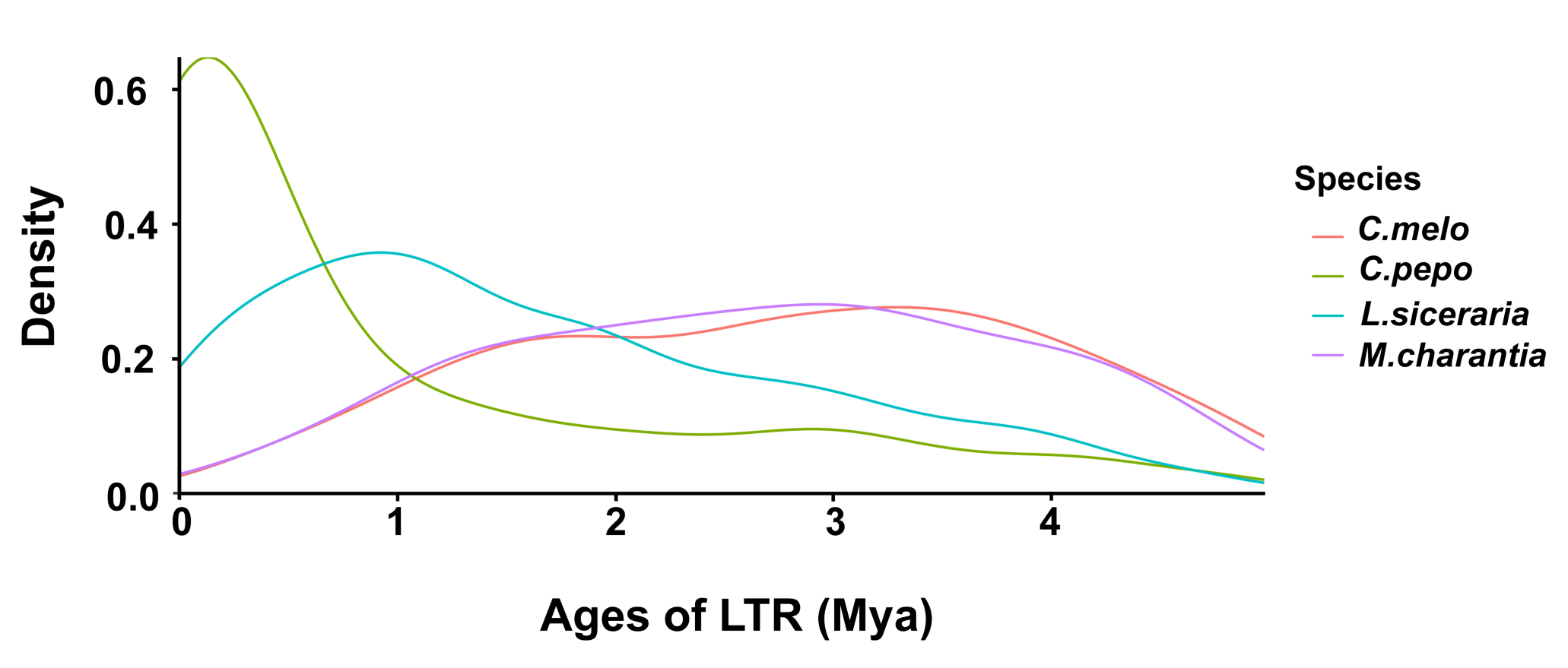


Figure S4 LTR insertion time analysis





Figure S5 Heat map of gene expression for plant hormone biosynthesis pathway and plant hormone signal transduction pathway. (A) Heat map of gene expression for auxin synthesis pathway. (B) Heat map of gene expression for ethylene synthesis pathway. (C) Heat map of gene expression for brassinosteroid synthesis pathway. (D) Heat map of gene expression for cytokinin synthesis. (E) Plant hormone signaling pathway gene expression heatmap.
